# Electronic Structures of Polymorphic Layers of Borophane

**DOI:** 10.3390/molecules27061808

**Published:** 2022-03-10

**Authors:** Ikuma Tateishi, Xiaoni Zhang, Iwao Matsuda

**Affiliations:** 1 RIKEN Center for Emergent Matter Science, Wako 351-0198, Saitama, Japan; 2 Institute for Solid State Physics, The University of Tokyo, Kashiwa 277-8581, Chiba, Japan; xnzhang17@g.ecc.u-tokyo.ac.jp (X.Z.); imatsuda@issp.u-tokyo.ac.jp (I.M.)

**Keywords:** borophene, borophane, 2D material, topological material, crystalline symmetry, Dirac electron, Dirac nodal line material

## Abstract

The search for free-standing 2D materials has been one of the most important subjects in the field of studies on 2D materials and their applications. Recently, a free-standing monolayer of hydrogenated boron (HB) sheet has been synthesized by hydrogenation of borophene. The HB sheet is also called borophane, and its application is actively studied in many aspects. Here, we review recent studies on the electronic structures of polymorphic sheets of borophane. A hydrogenated boron sheet with a hexagonal boron frame was shown to have a semimetallic electronic structure by experimental and theoretical analyses. A tight-binding model that reproduces the electronic structure was given and it allows easy estimation of the properties of the material. Hydrogenated boron sheets with more complicated nonsymmorphic boron frames were also analyzed. Using the symmetry restrictions from the nonsymmorphic symmetry and the filling factor of hydrogenated boron sheets, the existence of a Dirac nodal line was suggested. These studies provide basic insights for research on and device applications of hydrogenated boron sheets.

## 1. Introduction

In recent years, two-dimensional (2D) materials have attracted much interest due to their unique physical properties and potential applications in a variety of quantum devices [1,2,3,4,5]. One of the most famous 2D material is graphene. Graphene shows a wide variety of interesting properties, such as a high mobility, the anomalous integer quantum Hall effect, edge-dependent mesoscopic effect, and so on [6]. In particular, it is known that the linear dispersive electronic band of graphene, which is called Dirac electron, and the topological properties of the wave function around it play an important role in the unique physical properties of graphene. Recently, as an analog of graphene, honeycomb-lattice materials with other atoms (Xenes) such as silicene [7,8,9,10,11], phosphorene [12,13,14,15,16], germanene [11,17,18,19], arsenene [14,20,21], antimonene [14,20,22], and bismuthene [14,23,24], have also been actively studied. These materials are expected to be a new platform to explore unique physical properties qualitatively different from those of graphene, taking advantage of the difference in model parameters such as the strength of spin–orbit interaction. As a development in another direction, a material with a similar topological electronic band structure has been explored. The typical example is a Dirac nodal line material [25,26,27]. While graphene has a band dispersion with degenerate points with a linear dispersion around it, so-called Dirac points and Dirac cones, Dirac nodal line materials have a degenerate line in the momentum space in the band dispersion. Many nodal line materials have been proposed in 3D crystals [27,28,29,30,31,32], and Cu${}_{2}$Si [5] and CuSe [33] are also known as 2D Dirac nodal line materials. While these 2D materials possess attractive properties, most of them are restricted to preparation on substrates. For convenience in experiments and applications, the search for free-standing 2D materials has been one of the important subjects in this field [34]. Very recently, a monolayer of boron sheet has been synthesized by hydrogenation of a borophene frame in a MgB${}_{2}$ crystal [35,36], which is one of the Xenes. The obtained monolayer hydrogenated boron (HB) sheet, or “borophane”, has been attracting attention as a new 2D material [37,38,39,40]. Because the HB sheets are synthesized from boron frames in a 3D crystal material, another type of HB with a different boron frame has also been synthesized [41]. Not only a variety of borophene systems synthesized on metal substrates [42,43,44,45,46,47], but the free-standing HB sheets are also an important platform to study the 2D boron materials.

In this review, we first review what kind of boron frame we can find in 3D crystals that exist in nature. Next, we review recent works on the electronic structure of a HB sheet with a honeycomb boron frame, which is the most basic example of HB. Based on that, we present two subsequent studies. One of them is a study on the electronic structure of honeycomb HB nanoribbons. The other is a study on HB sheets with other types of boron frame, nonsymmorphic HB sheets. Finally, we review the results of these experimental studies on the HB sheets.

## 2. Borophene in Natural 3D Crystals

The synthesis of a HB sheet is realized by the proton ion-exchange reaction of magnesium diboride (MgB${}_{2}$): MgB${}_{2}$ + 2H${}^{+}$ → Mg${}^{2+}$ + 2HB [35]. By this method, the honeycomb boron frame in MgB${}_{2}$ is peeled and obtained as a free-standing monolayer with the hydrogen termination. This method can be used in other metal-boron-layered materials to obtain other types of HB sheets. Therefore, in this section, we first review what kind of borophene layer we can find in 3D crystals in nature.

**Figure 1:**
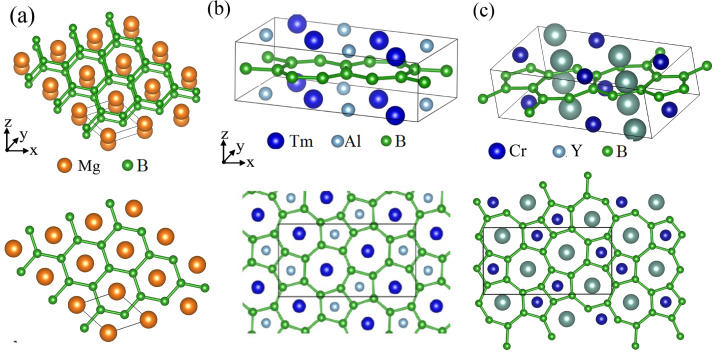
Crystal structures of metal-boron-layered materials. (**a**) Crystal structure of MgB${}_{2}$. (**b**) Crystal structure of TmAlB${}_{4}$. (**c**) Crystal structure of YCrB${}_{4}$.

The most standard one is the hexagonal boron frame in MgB${}_{2}$ [48] (Figure 1a), as introduced above. In a MgB${}_{2}$ crystal, there are two layers in the unit cell, a Mg layer and a hexagonal borophene layer. It is noteworthy that the borophene layer has the same symmetry as graphene. Because graphene exhibits a great variety of quantum phenomena, the hexagonal borophene and HB from MgB${}_{2}$ is the most actively studied material in the field of borophene and HB.

The next example is TmAlB${}_{4}$ [49], which consists of a metal atoms (Tm,Al) layer and a borophene layer (Figure 1b). The characteristic feature of this material is that the crystal and borophene frame have a nonsymmorphic symmetry. A nonsymmorphic symmetry is a symmetry described by a screw or glide, which are a rotation or mirror followed by a fractional translation, respectively. For example, when basic translation vectors $a_{1}$, $a_{2}$, and $a_{3}$ are, respectively, defined along the *x*, *y*, and *z* axes, the crystal structure of TmAlB${}_{4}$ is invariant for a screw $\left\{C_{2x}\left|\frac{1}{2}\frac{1}{2}0\right.\right\}$, which is a two-fold rotation along the *x* axis ($C_{2x}$) followed by a fractional translation $\frac{1}{2}\left(a_{1}+a_{2}\right)$. Because this nonsymmorphic symmetry gives a strong restriction on the electronic band structure of the material, the borophene and HB from TmAlB${}_{4}$ is also an interesting target of research in this field. The same borophene frame is also found in YCrB${}_{4}$ (Figure 1c) [50]. These materials can be used as parent materials of nonsymmorphic borophene and HB.

In the following sections, we see electronic structures of the borophene and HB obtained from these materials. First, we review a study on the most standard ones, hexagonal borophene and HB.

## 3. HB Sheet with Hexagonal Boron Frame

**Figure 2:**
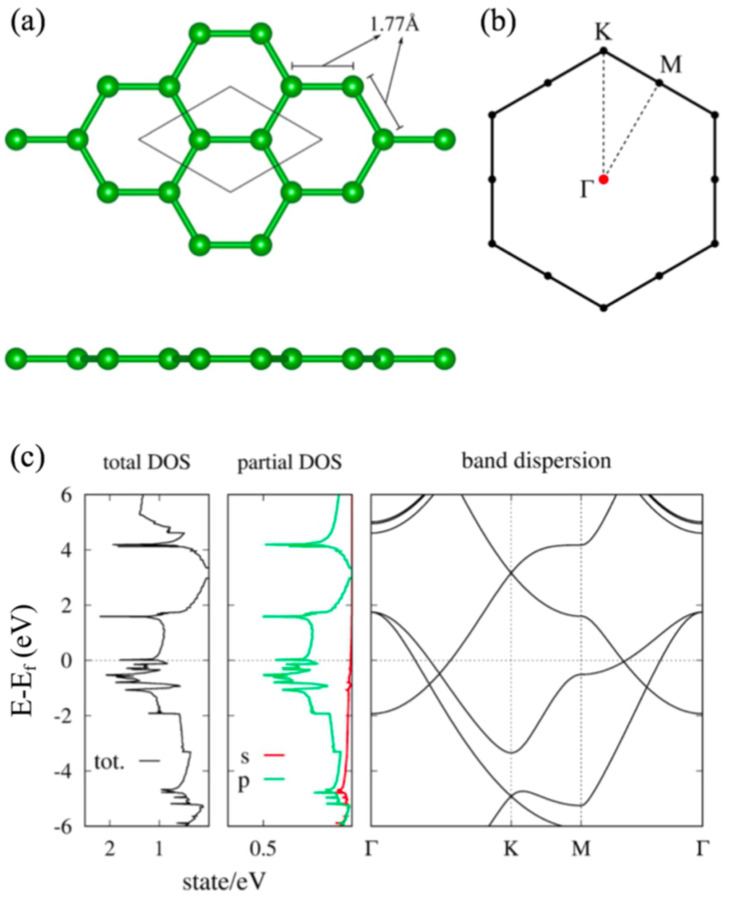
(**a**) Lattice structure and (**b**) BZ of the honeycomb boron sheet. (**c**) Density of states and electronic band dispersion of the honeycomb boron sheet (adapted from [37]).

In this section, we review the hexagonal borophene and HB sheet [37]. As easily expected, a hydrogenation generally has a significant impact on the electronic structure [51,52]. The first question to be considered is how this hydrogenation affects the electronic structure in the hexagonal HB sheet. In particular, the presence or absence of topologically nontrivial band structures such as graphene is of interest. The Dirac electron at the K point of graphene is attributed to the symmetry of the honeycomb structure of graphene and its filling factor. Because the hexagonal borophene from MgB${}_{2}$ has the same structure as graphene, the electronic band structure is really similar to that of graphene but the Fermi energy is not located on the Dirac point. Figure 2 is the electronic structure of borophene obtained by the first-principles calculation (GGA-PBE exchange correlation functional). We can see there is a Dirac electron around 3 eV at the K point as in graphene. Since boron has fewer electrons than carbon, the Dirac cone is located above the Fermi level.

**Figure 3:**
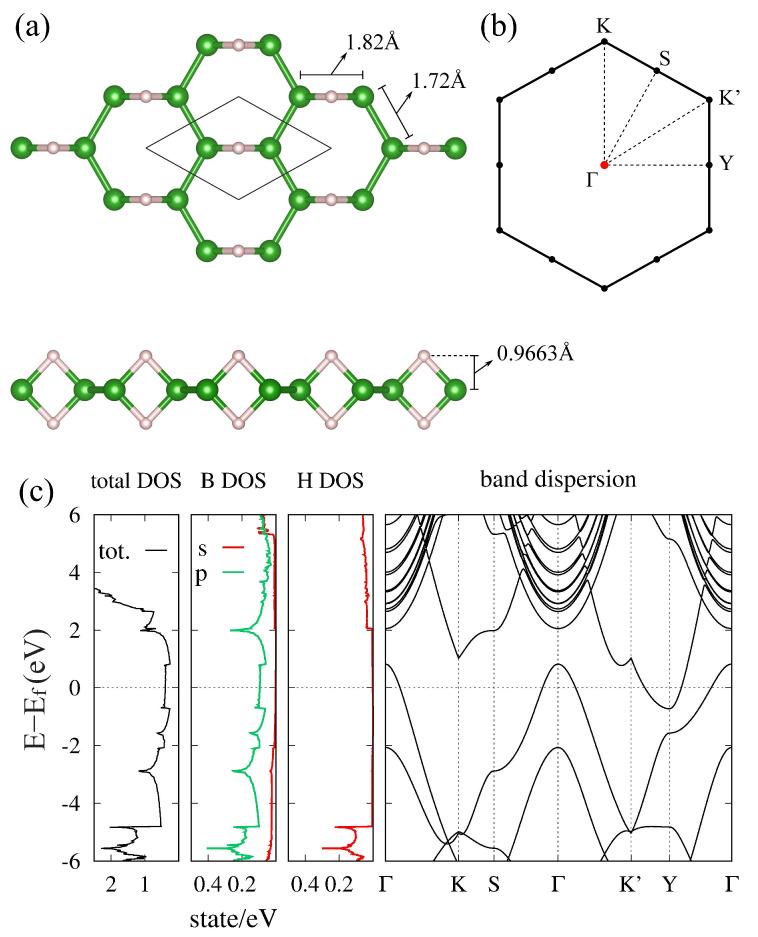
(**a**) Lattice structure and (**b**) BZ of the hexagonal HB. (**c**) Density of states and electronic band dispersion of the hexagonal HB (adapted from [37]).

When the borophene is modified into HB, the honeycomb symmetry is broken by hydrogenation. Therefore, the electronic structure of the hexagonal HB sheet should be analyzed in detail. Figure 3 is the electronic structure of the hexagonal HB sheet. The electronic band structure of the hexagonal HB sheet (Figure 3) does not resemble that of the honeycomb boron sheet and Dirac electrons are no longer found. We can see that the hexagonal HB sheet is a semimetal with a hole pocket at the Γ point and an electron pocket at the Y point in the Brillouin zone (BZ). The effective mass of the carrier is estimated as $\left(m_{xx}^{\Gamma },m_{yy}^{\Gamma }\right)≃\left(-4.02m_{e},-2.23m_{e}\right)$ for a hole and $\left(m_{xx}^{Y},m_{yy}^{Y}\right)≃\left(1.65m_{e},11.4m_{e}\right)$ for an electron, respectively ($m_{e}$ is the bare electron mass). In [37], it was shown that the electron and hole pockets originate from some electronic bands in the honeycomb boron sheet by varying the position of the hydrogen and checking the symmetry of the wave functions. As a result, it was found that the hole pocket originates from one of the doubly degenerate hole pockets at the Γ point in the honeycomb boron sheet, while the electron pocket originates from the band around 2 eV at the M point.

From this result, the effects of hydrogenation and the B–H–B bond on the electronic structure can be generally discussed. Although the symmetry and electronic structure are significantly changed by hydrogenation, the bonding and antibonding states of the B–H–B bond appear in the energy region far from the Fermi level, and thus there is almost no density of state (DOS) of hydrogen near the Fermi level (Figure 3). As a result, the electronic states derived from the boron frame remain around the Fermi level even after hydrogenation. This allows partial inheritance of the properties of the boron frame before hydrogenation. For example, the possibility of two-dimensional superconductivity is pointed out in [37]. Although not all the properties are necessarily inherited from the boron frame because the electronic state is significantly changed by hydrogenation, it may provide potential applications for hydrogenated boron sheets.

A tight-binding model of the hexagonal HB sheet has also been proposed in [37]. The basis of the tight-binding model consist of $p_{z}$ orbitals on the boron atoms and bonding states of the B–B bonds. In [37], the electronic structure has been qualitatively discussed only with the nearest-neighbor hoppings, and it is shown that hopping along the B–H–B bond is larger than others. This tight-binding model can be used as a convenient tool for the analysis of the HB sheet.

Based on the results of the analysis on the hexagonal borophene and HB, here we show two ways to extend the study on borophene and HB. The first one is aimed at nanodevice applications of the hexagonal HB. We show electronic structures of HB nanoribbons in Section 4 as an example. The other way is an investigation of topological bands that are given by the nonsymmorphic symmetry in other HB sheets, which is reviewed in Section 5.

## 4. HB Nanoribbon

**Figure 4:**
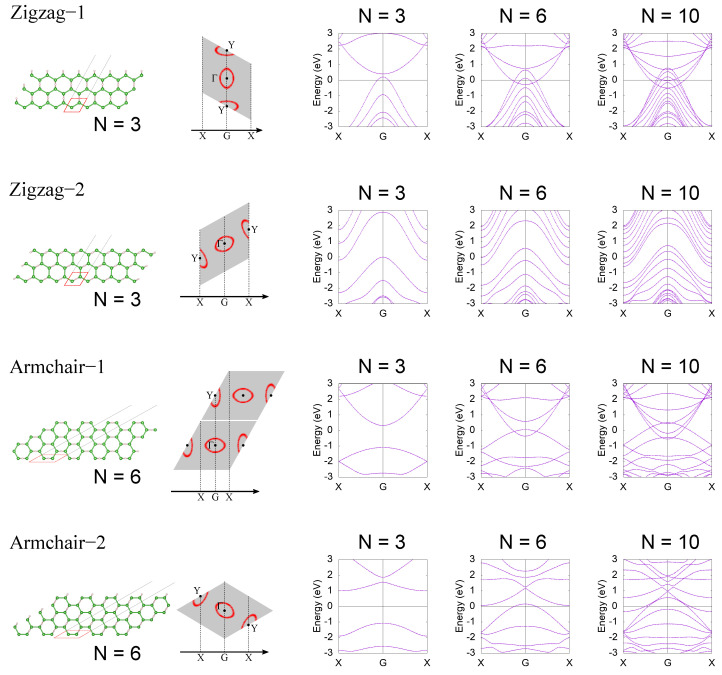
HB nanoribbon and its electron structure. Red cells in lattice structure figures are the unit cells that construct the nanoribbon. In each row (each truncation type), the definition of the 1D BZ is shown with the 2D BZ (gray shaded rhombus) in the second right figure. For four edge truncation types, electron band structures of nanoribbons are calculated for a three-unit cell case (N=3), a six-unit cell case (N=6), and a ten-unit cell case (N=10).

In this section, we calculate the electronic structure of a hexagonal HB nanoribbon. To discuss the anisotropy and the spreading of the wave function due to the H–B–H coupling in detail, we used the same calculation method as in [37], but including the long-range hoppings. The tight-binding model was obtained by Wannier90 [53], and electronic band structures of nanoribbons are calculated using the WannierTools package [54]. We considere four edge truncation types, zigzag-1 (ZZ1), zigzag-2 (ZZ2), armchair-1 (AC1), and armchair-2 (AC2) shown in Figure 4. We calculate the electronic structures of the four types of nanoribbons by changing the ribbon width. The ribbon width is given as a number of unit cell (*N*), and the unit cell is shown as a red dashed cell in the left figure of each row in Figure 4. The 2D BZ (gray shaded rhombus) and corresponding 1D BZ of each nanoribbon are shown in the second column of each row. The red lines in the 2D BZ are Fermi surfaces of the electron and hole pockets. The right three panels are electronic band structures of the nanoribbon for N=3, N=6, and N=10. In the case of ZZ1, the electron and hole pockets overlap at the G point, and the system becomes a 1D conductor when the ribbon width is large enough (N=10). As the ribbon width gets smaller, the electronic state is modified by a finite size effect that comes from a spread of the Wannier functions. Around N=3∼6, the finite effect becomes significant and the electronic structure becomes gapped in N=3. This result indicates that hoppings between the third nearest cells have a non-negligible effect on the low-energy electronic structure. In the case of ZZ2, the hole pocket is projected on the G point while the electron pocket is projected on the X point in the 1D BZ. Therefore, the electronic structure of the ZZ2 nanoribbon is semimetallic. Furthermore, in the ZZ2 case, the ribbon width dependence is almost the same as that of the ZZ1 case, and the electronic structure becomes gapped around N=3. Next, we move to the AC1 and AC2 cases. In the AC1 case, the electronic structure in a large ribbon width case is metallic, as in the case of ZZ1. The AC2 case is in principle semimetallic, but the electron and hole pockets are so large that the system becomes metallic in a large ribbon width case as N>10. In the cases of AC1 and AC2, the finite size effect is significant in N<6∼10. This is simply because the unit cells that make up the AC1 and AC2 nanoribbons are heavily distorted and their width is small compared to the case of ZZ1 and ZZ2. Note that the ribbon widths of the N=6 ribbons in the AC1 and AC2 cases are comparable to those of the N=3 ribbons in the ZZ1 and ZZ2 cases (roughly 8 Å). These results are consistent with the first-principles calculations in [55].

In the above, as an example of using the tight-binding model, we have calculated the electronic structure of HB nanoribbons and their ribbon width dependence. In the small ribbon width case, the finite size effect gets significant and the electronic structure becomes gapped. When the ribbon width is smaller than 7 Å, the HB nanoribbon is expected to be a 1D small-gap semiconductor. By changing the edge truncation, one can choose a direct or indirect gap.

## 5. HB Sheet with Nonsymmorphic Boron Frame

In this section, we review the electronic structures of nonsymmorphic HB sheets from TmAlB${}_{4}$ or YCrB${}_{4}$ [38]. In the nonsymmorphic HB sheets, the existence of a Dirac nodal line has been proposed. First, we explain the symmetry restrictions on the electronic band structure given by the nonsymmorphic symmetry. Because the nonsymmorphic operation is combined with a fractional translation, a quasi-band-folding is generally seen in the band structure. As a result, these nonsymmorphic operators generally guarantee double degeneracies of electronic bands on some parts of the BZ boundary. If the band folding is given by a pure fractional translation, all momenta k are invariant for the pure translation and thus an eigenvalue of the pure translation is defined for all Bloch wave functions $\psi_{k}$. Even if a Dirac nodal line is obtained by the band folding, an optical excitation between bands with different translation eigenvalues is basically prohibited, and thus the obtained Dirac nodal line is not usually used as a linear-dispersive band. On the other hand, the nonsymmorphic operations are combined with rotations or mirrors, and thus a generic momentum k is not invariant for the operation. Therefore, the Dirac nodal line obtained by the quasi-band-folding of nonsymmorphic symmetry can be used as a linear-dispersive band.

**Figure 5:**
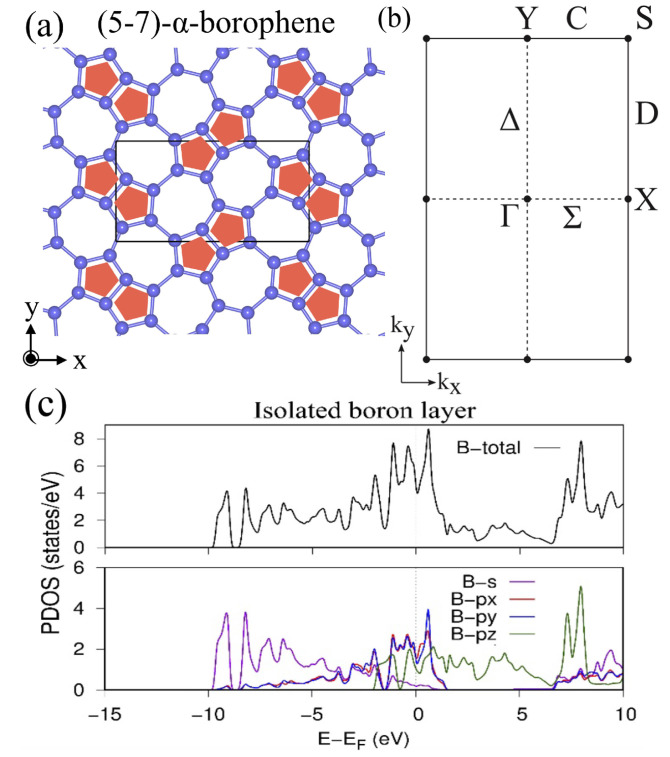
(5–7)-α-borophene. (**a**) Lattice structure of (5–7)-α-borophene. (**b**) BZ of the (5–7)-α-borophene. (**c**) Total and partial density of state of (5–7)-α-borophene obtained by the first-principles calculation (adapted from [38,41]).

The nonsymmorphic boron frame obtained from TmAlB${}_{4}$ is named as (5–7)-α-borophene (Figure 5), which belongs to the layer group Pbam (layer group no. 44, or 3D space group no. 55). Here, (5–7)-α indicates that the α lattice is composed of pentagons and heptagons. Especially in this layer group, it is proved that the double degeneracy given by the nonsymmorphic symmetry occurs on all points on the BZ boundary. This general symmetry restriction contributes to the appearance of a Dirac nodal line when the filling factor of the HB sheet is additionally considered. Generally, the ratio of B and H atoms is always B:H = 1:1. Further, the number of B atoms in the unit cell in a Pbam symmetric system is an even number due to the multiplicity from the nonsymmorphic operation. As a result, the filling factor (number of electrons in the unit cell) is written as $12n=4n^{\prime }$, where *n* and $n^{\prime }$ are integers. Because each doubly degenerate band on the BZ boundary consists of four states, the lower $n^{\prime }$ degenerate bands are completely occupied and the others are completely empty. These restrictions on the band degeneracy and its filling play an important role to determine the presence or absence of the Dirac nodal line in the material. By using this restriction, in [38], a mirror-eigenvalue-based index has been proposed to determine the presence or absence of a Dirac nodal line. Note that the well-known inversion-based index (Fu–Kane index [26,56]) does not correctly work in 2D systems. It is noteworthy that one needs to check wave functions only at the Γ point to calculate the mirror-eigenvalue-based index. Generally, in a 2D system, a possible Dirac nodal line is always protected by a mirror symmetry on the xy plane and thus the presence or absence is determined by checking the parity of the number of occupied bands that have a mirror eigenvalue −1 in each momentum. In [38], it was proved that the method gets even easier especially in the layer group Pbam with a filling factor $4n^{\prime }$. By the representation theory of the layer group Pbam, it is proved that the doubly degenerate bands on the BZ boundary always have the same mirror eigenvalue. As a result, the number of occupied bands with a mirror eigenvalue −1 on the BZ boundary must be an even number. Due to this symmetry restriction on the BZ boundary, one only needs to check the parity at the Γ point to determine the presence or absence of a Dirac nodal line. When the number of occupied bands with a mirror eigenvalue −1 at the Γ point is odd, there is a Dirac nodal line and thus the material is a Dirac nodal line semimetal. Among all 80 layer groups, the layer groups Pbam (no. 44) and P4/mbm (no. 64) have these properties.

**Figure 6:**
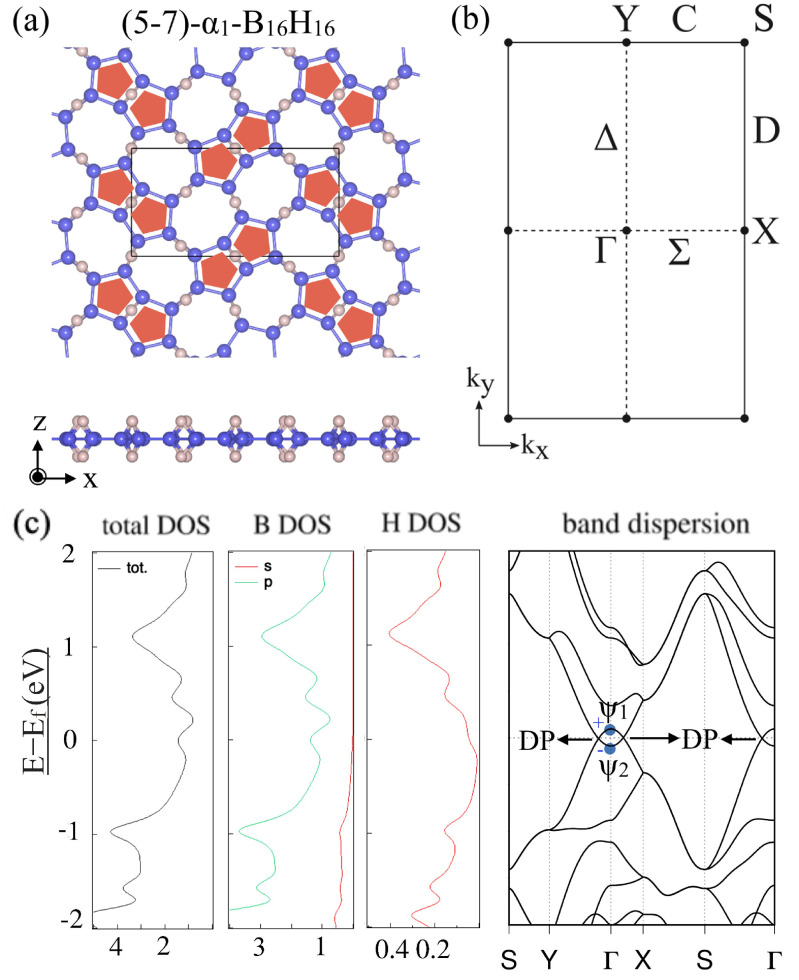
(5–7)-$\alpha_{1}$-HB. (**a**) Lattice structures of (5–7)-$\alpha_{1}$-HB. (**b**) BZ of (5–7)-$\alpha_{1}$-HB. (**c**) Electronic band structure and density of state of (5–7)-$\alpha_{1}$-HB. The Dirac points (DP) are parts of the Dirac nodal line (adapted from [38]).

**Figure 7:**
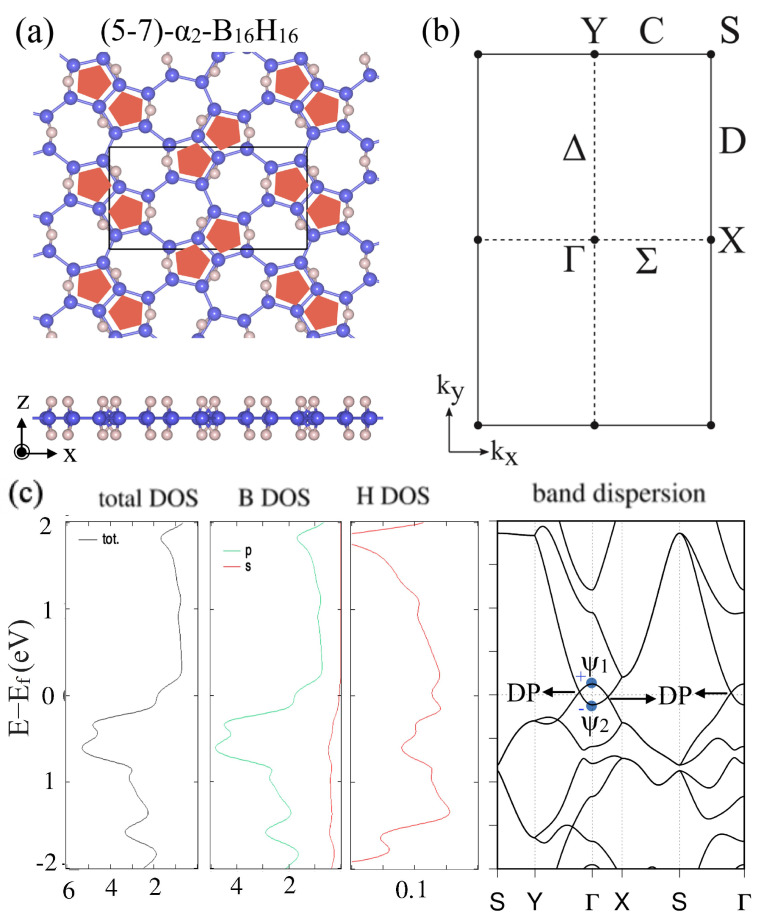
(5–7)-$\alpha_{2}$-HB. (**a**) Lattice structures of (5–7)-$\alpha_{2}$-HB. (**b**) BZ of (5–7)-$\alpha_{2}$-HB. (**c**) Electronic band structure and density of state of (5–7)-$\alpha_{2}$-HB. The Dirac points (DP) are parts of the Dirac nodal line (adapted from [38]).

After this general discussion, the electronic band structures and appearance of a Dirac nodal line were specifically shown for two types of HB sheets with different hydrogenation patterns for the α-borophene frames. Both of $\alpha_{1}$ and $\alpha_{2}$ were predicted to be Dirac nodal line semimetals (Figure 6 and Figure 7). Figure 6 shows (a) the lattice structure, (b) the BZ and high-symmetry points and lines, (c) the total and partial density of states and the band dispersion of the (5–7)-$\alpha_{1}$-HB. Figure 7 shows those of the (5–7)-$\alpha_{2}$-HB. Note that the electronic bands on the BZ boundary (the S–Y line and S–X line) are doubly degenerate due to the symmetry restriction explained above. The mirror eigenvalues of the wave functions are also shown in Figure 6c and Figure 7c. Because a band inversion between two bands with the opposite mirror eigenvalues occurs at the Γ point, the number of occupied bands with a mirror eigenvalue −1 at the Γ point is odd in these cases. We can see there are Dirac points (DP) on the Γ–Y and Γ–X lines, which are parts of the Dirac nodal line. Not only do these materials have Dirac nodal lines, but there is also no band other than the Dirac nodal line around the Fermi level and thus they are ideal 2D Dirac nodal line semimetals. Although an easy model has not been given in [38], the bonding types are almost identical with the hexagonal HB case and thus the finite size effect in a nanoribbon is expected to appear in the same ribbon width.

In the above, we have reviewed the electronic band structures of (5–7)-α-HB sheets that are obtained from TmAlB${}_{4}$. Due to the symmetry restriction in the band structure and filling factors of the systems, these materials were predicted to be good examples of 2D Dirac nodal semimetals. In [38], another type of nonsymmorphic HB sheet, (5,6,7)-γ-HB, and the existence of a Dirac nodal line were also discussed. Additionally, even if a boron frame is symmorphic, the hydrogenation pattern can break the symmetry of the boron frame and consequently a nonsymmorphic HB sheet can be obtained. As we have seen in this section, the nonsymmorphic HB sheets have a potential as a topological material while the hexagonal HB sheet does not have a topological band.

## 6. Experiments and Applications

Finally in this section, we review experimental studies of HB sheets and application studies.

**Figure 8:**
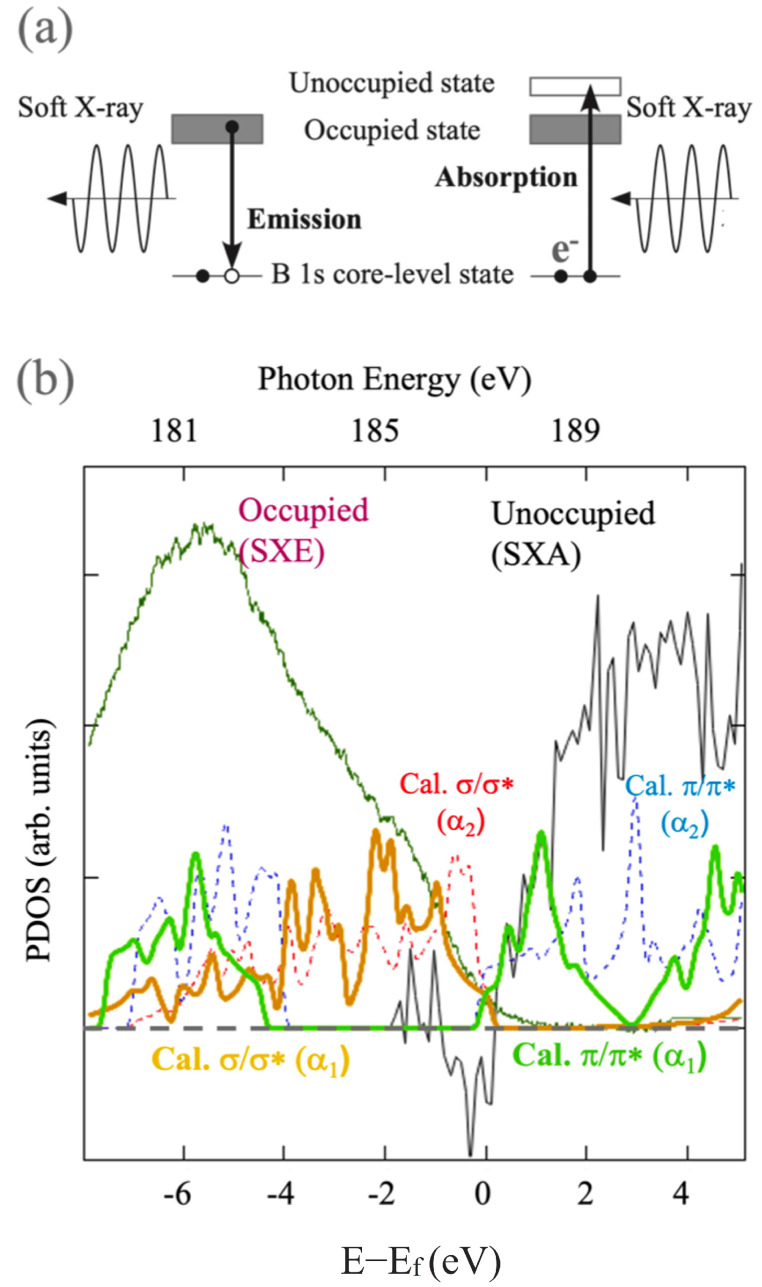
X-ray spectroscopy experiments on HB sheets. (**a**) Schematic picture of the soft X-ray emission and absorption (SXE and SXA) spectroscopy. (**b**) Spectroscopy data of the (5–7)-α HB sheet and calculated σ/σ* and π/π* bands for the (5–7)-$\alpha_{1}$ and (5–7)-$\alpha_{2}$ HB sheets (adapted from [41]).

The electronic structures of HB sheets have also been studied by X-ray spectroscopy experiments [37,41]. Both of the hexagonal and (5–7)-α HB sheets have been confirmed to be semimetallic at least with power samples. For example, the spectroscopy data of the (5–7)-α HB sheet is shown in Figure 8. The HB sheet was synthesized by the proton ion-exchange method, prepared by liquid exfoliation for measurement [35]. The spectra of the soft X-ray emission and absorption (SXE and SXA) at B K-edge were conducted in the BL-09A at the NewSUBARU Synchrotron Radiation Facility [57]. The spectroscopy measurements were carried out at room temperature. The calculated σ/σ* and π/π* bands for the (5–7)-$\alpha_{1}$ and (5–7)-$\alpha_{2}$ HB sheets are also shown for comparison. It is of note that the SXE and SXA spectra indicate the dipole transition between 2p and 1s states, reflecting the occupied valence band and unoccupied conduction band, respectively, [41,49], as shown in Figure 8. One can find that the spectroscopy results agree well with calculated bands, especially with the α1 type. The consistent result provides evidence of semimetallicity and implies the Dirac nodal loop exists near the Fermi level.

Lastly, we mention the potential for other applications. The hexagonal HB has been studied for applications as catalysts [58,59,60,61,62] and hydrogen storage [63,64]. As a catalyst, an ethanol–ethylene conversion mechanism on the HB sheet has been studied in [59]. As hydrogen storage, decorated HB nanotubes have analytically been studied in [64], and a photoinduced hydrogen release has been experimentally reported in [63].

## 7. Summary and Outlook

We reviewed recent studies on electronic structures of free-standing monolayer hydrogenated boron (HB) sheets. HB sheets have recently been synthesized by the proton ion-exchange reaction and have been attracting attention as a new 2D material in both experimental and theoretical studies. A hexagonal hydrogenated boron sheet, which is synthesized from MgB${}_{2}$, was revealed to have a semimetallic electronic structure. A tight-binding model that reproduces the electronic state was given and it allowed us to easily estimate the properties of the system, for example, the electronic band structures of nanoribbons. Nonsymmorphic hydrogenated boron sheets, which are synthesized from TmAlB${}_{4}$, were also proposed as 2D materials that host Dirac nodal lines around the Fermi level. The appearance of the Dirac nodal line was strongly related to the symmetry restrictions on the band dispersion. The semimetallic electronic structure or the existence of the Dirac nodal line was also experimentally confirmed. In the future, it is expected that large sheet samples of hydrogenated boron sheets will be obtained that will allow a more detailed electronic structure analysis and application studies. In addition, if it becomes possible to synthesize more diverse HB sheets, it is expected that we will be able to select and use an ideal HB sheet as a 2D material for various applications.

## Data Availability

No datasets were generated during the current study.
